# Swedish national cohort of children living with long-term respiratory support (DISCOVERY-P): cohort profile

**DOI:** 10.1136/bmjopen-2024-090241

**Published:** 2025-04-14

**Authors:** Johan Florén, Magnus Ekström, Berit Lindahl, Agneta Markström, Andreas Palm, Åsa Israelsson-Skogsberg

**Affiliations:** 1 Faculty of Caring Science, University of Borås, Borås, Sweden; 2 Department of Clinical Sciences, Division of Respiratory Medicine & Allergology, Lund University, Lund, Sweden; 3 Department of Medical Sciences, Lung- allergy- and sleep research, Karolinska University Hospital, Stockholm, Sweden; 4 Department of Medical Sciences, Respiratory, Allergy and Sleep Research, Uppsala University, Uppsala, Sweden

**Keywords:** Retrospective Studies, Child, Adolescent, Risk Factors, Chronic Disease, RESPIRATORY MEDICINE (see Thoracic Medicine)

## Abstract

**Abstract:**

**Purpose:**

Children living with respiratory support rely on medical technology, either fully or partially, throughout the day to meet their breathing requirements. Although children and young people living with respiratory support at home undergo long-term treatments and make extensive use of health and social care services, there is a notable absence of comprehensive outcome data on this group. The establishment of the first nationwide Course of DISease reported to the Swedish CPAP Oxygen and VEntilator RegistrY paediatrics cohort aims to investigate the disease trajectory, clinical and socioeconomic risk factors influencing incident illness, hospitalisation risk and mortality among children living with respiratory support.

**Participants:**

Data on patients aged 0–18 years reported to the Swedish National Registry for Respiratory Failure and Sleep Apnoea (Swedevox) 1 January 2015 to 29 July 2021 were merged with seven quality or governmental registries, the National Quality Registry for Intensive Care, the National Medical Birth Register, the Swedish Cause of Death Registry, the Registry for Interventions under the Act on Support and Service to Certain Disabled Persons, the Swedish National Patient Registry and with socioeconomic data from Total Population Registry and Longitudinal Integrated Database for health insurance and labour market studies.

**Findings to date:**

The cohort includes 716 children, 59% male, who began respiratory support at an average age of 6.4 years (SD 5.4). Among them, 28% use continuous positive airway pressure, 64% long-term mechanical ventilation (LTMV), 3% high-flow oxygen therapy (HFOT) and 5% other methods. Respiratory support is mostly used at night, but many LTMV (54%) and HFOT (81%) users need daytime aid. 77% of LTMV users rely on mask connection, differing from international data.

**Future plans:**

Future projects include exploring the impact of socioeconomic factors on hospitalisation rates and mortality. The dataset is due for an update in 2026.

**STRENGTHS AND LIMITATIONS OF THIS STUDY**

- This nationwide population-based cohort stands out for its competitive size, extensive nationwide representation and remarkable data completeness for included individuals.
- No registered patients are lost to follow-up due to cross-linkage with mandatory national registries.
- Distinctive database to analyse longitudinal data from diverse sources from medical outcomes to socioeconomic factors.
- National quality registries (Swedevox) lack mandatory status, resulting in somewhat varying coverage.

## Introduction

 Children (aged 0–18 years) who require respiratory support rely on medical technology either entirely or partially throughout the day to fulfil their breathing needs. Advanced technology, such as continuous positive airway pressure (CPAP), long-term mechanical ventilation (LTMV) and long-term high-flow oxygen therapy (HFOT), along with comprehensive treatment and care enables them to live active and healthy lives and engage in societal activities.^1^ However, these individuals have overcome conditions that were once life-threatening or required prolonged hospitalisation. They now depend on functional technology, support from their families and communities and personal care assistance to maintain their respiratory and overall health.^2 3^ Under legislation in Sweden,^4^ children who use respiratory support can access individually tailored personal care assistance.^5^

Sweden has a long history of keeping thorough records of its inhabitants. There are, in total, 107 national quality registers containing individual data concerning different diagnoses and treatments.^6^ The registers related to health and social services are managed by the National Board of Health and Welfare (Socialstyrelsen),^7^ and Statistics Sweden (SCB) manages registries related to population data.^8^ The use of common personal identity numbers^9^ allows identification and cross-linkage across the different registers, thus supporting population-based outcome studies.

In Sweden, around 700 children are currently registered with the National Registry for Respiratory Failure and Sleep Apnoea (Swedevox), indicating their reliance on respiratory support while residing at home.^10^ In recent decades, there has been a rise in the number of new users requiring respiratory support globally.^211^,^13^ Though there are quantitative studies done internationally concerning the group,^13^ there is a lack of studies focusing on socioeconomic factors as well as geographical concerns. This population is diverse, and the underlying reasons for their respiratory support needs vary. Many children have multiple underlying conditions that further complicate their circumstances, highlighting the need for personalised solutions.^2 14^ Consequently, there is a large need for professional care in supervising these children, helping them with wheelchairs and tube feeding them at all hours of the day.^15^

Respiratory insufficiency can be caused by factors such as neuromuscular diseases, brain and spinal cord injuries, lung tissue damage and central hypoventilation.^11 16^ Previous studies using data from children living with respiratory support across different regions globally^16^,^20^ have shown regional variations in practices and outcomes influenced by factors such as healthcare infrastructure and socioeconomic conditions. In the Swedish context, the population has been previously examined through qualitative methodologies^121^,^27^ indicating the possibilities that this group can live good lives even though the effects of the condition on freedom and interactions can be substantial. Although children and young people living with CPAP, LTMV and HFOT worldwide undergo long-term treatments and make extensive use of health and social care services, there is a paucity of large-scale outcome data available for this group overall and only a handful of small-scale cohorts describing such data from a Swedish perspective.^28^,^31^ Questions regarding comorbidity burdens, hospitalisation rates, mortality and treatment outcomes in children and youths living with CPAP, LTMV and LTHFO have been largely unexplored. Previously, data on adults reported to the Swedevox registry have been cross-linked with several governmental and quality registries, creating a research database, Course of DISease reported to the Swedish CPAP Oxygen and VEntilator RegistrY (DISCOVERY).^32^ Validation research based on Swedeox data confirms its quality and suitability for research purposes.^33^ We aimed to create a similar database for children reported to the Swedevox registry, DISCOVERY paediatrics (DISCOVERY-P). The objective of this cohort profile is to describe the design, data-assembling procedures and variables and to provide an overview of children living with respiratory support in Sweden, providing a base for further research.

## Cohort description

DISCOVERY-P is a nationwide population-based cohort aged 0–18 based on inclusion in the Swedish National Quality Registry for Respiratory Failure (Swedevox). Respiratory support included in Swedevox consists of CPAP and LTMV (both invasive and non-invasive); some children are registered as HFOT, or others, including tracheal cannula only and/or phrenic pacing. Data collection commenced after ethical approval had been obtained from the Swedish Ethical Review Authority (Dnr 2021–03426).

### Compilation of the database

Patients 0–16 years reported to the Swedevox registry’s paediatric arm between 1 January 2015 and 29 July 2021 were individually cross-linked with the following registries: the National Quality Registry for Intensive Care (SIR), National Medical Birth Register (MBR), the Swedish Cause of Death Registry (DORS), the Registry for Interventions under the Act on Support and Service to Certain Disabled Persons (LSS registry), the Swedish National Patient Registry (NPR), the Total Population Registry (RTB) and Longitudinal Integrated Database for Health Insurance and Labour Market Studies (LISA). Swedevox and the SIR are National Healthcare Registries independently administered by their own organisations, while DORS, the LSS registry and NPR are provided by the Swedish National Board of Health and Welfare. RTB and LISA are provided by Statistics Sweden (SCB).

**Figure 1:**
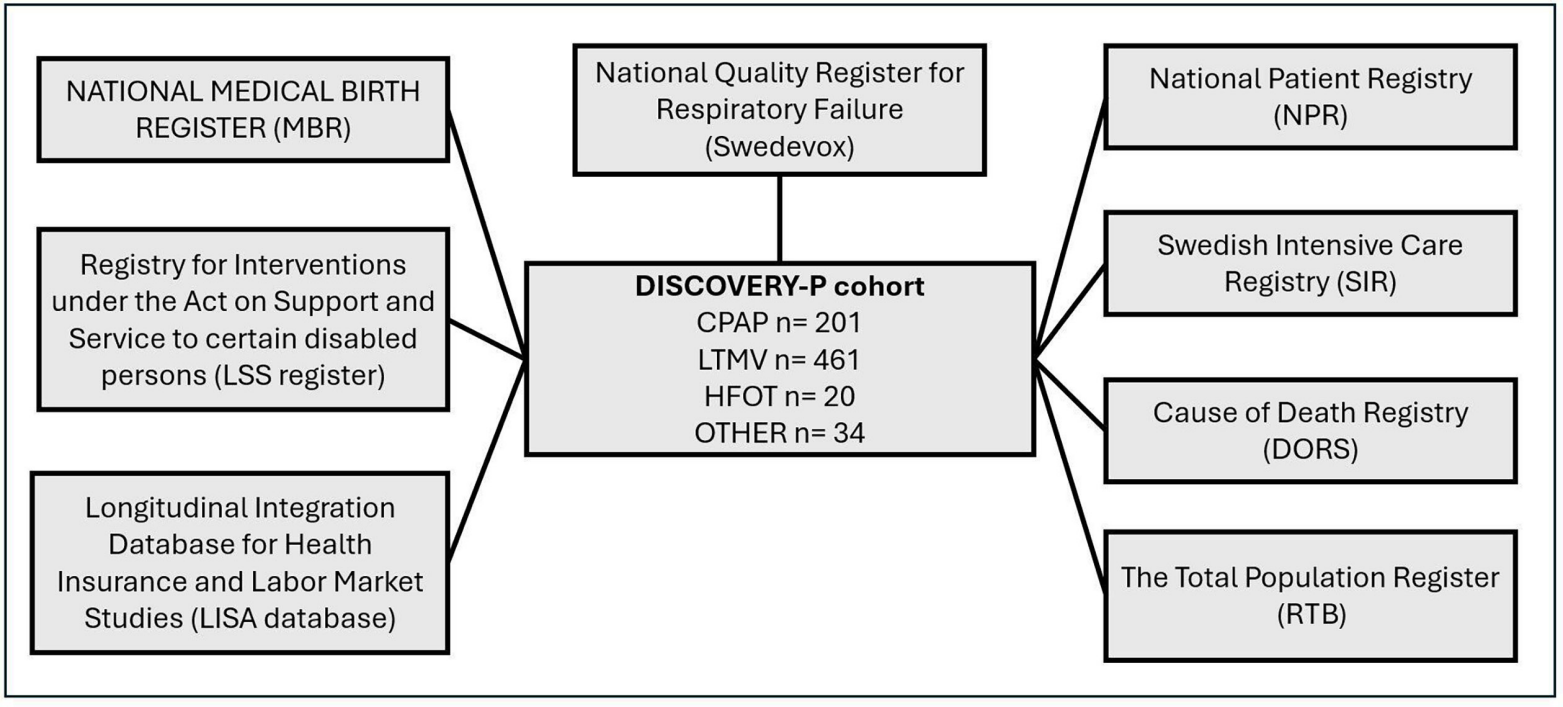
Overview of the eight registries contributing data to the Course of DISease reported to the Swedish CPAP Oxygen and VEntilator RegistrY paediatrics (DISCOVERY-P) cohort. Each registry is represented as a separate grey box, converging into a central box that represents the cohort. The cohort includes participant numbers categorised by treatment type. CPAP, continuous positive airway pressure; LTMV, long-term mechanical ventilation; HFOT, long-term high flow oxygen therapy.

The DISCOVERY-P dataset was compiled using data sourced from National Healthcare Registries, SCB and the Swedish National Board of Health and Welfare. Requests for data were sent to each registry keeper, who thereafter sent their respective data to the Swedish National Board of Health and Welfare, where it underwent pseudonymisation before being returned to the research group (figure 1).

Each individual in the registries was assigned a unique serial number by SCB and the Swedish National Board of Health and Welfare, streamlining the integration of various registries. The document containing personal identification numbers and corresponding serial numbers will be securely stored by the Swedish National Board of Health and Welfare for 3 years. This storage is essential to enable future corrections, follow-up data retrieval and the integration of additional registries into the DISCOVERY-P cohort.

#### Registries included in the Course of DISease reported to the Swedish CPAP Oxygen and VEntilator RegistrY paediatrics cohort

##### Swedish National Quality Registry for Respiratory Failure (Swedevox)

Patients have been included in the National Quality Registry for Respiratory Failure since 1987. The registry is divided into four different arms: the long-term oxygen therapy arm starting in 1987, the LTMV arm starting in 1996, the CPAP arm starting in 2010 and the children’s arm starting in 2015.^32^

Before 2015, children were not separated from adults in the registry, which was problematic because diagnosis and treatment strategies differ between adults and children. Since 2015, the diagnoses and treatments included in the registry have been adapted to the paediatric population. The maximum age for children to be included in the children’s arm of the registry is 18 years old, but new admissions to the registry are only accepted up to 16 years of age at the initiation of treatment. As individuals reach this age, the spectrum of diseases they experience starts to resemble that of adults. Diagnoses and groupings reported to the Swedevox children’s arm are brain diseases, including cerebral paresis and other brain damage (congenital or acquired); congenital central hypoventilation syndrome; restrictive thoracic diseases, including scoliosis and arthrogryposis; lung diseases, including pulmonary hypoplasia after diaphragmatic hernia; sleep apnoea, alone or as part of another disease, including craniofacial malformations and Mb Down; neuromuscular diseases, including spinal muscular atrophy type II–III–IV and Duchenne muscular dystrophy; and other non-specified diagnoses. The patients have scheduled annual follow-ups that are registered in Swedevox for the first 3 years. Table 1 presents the list of variables reported to the registry.

**Table 1:** Swedevox variables at baseline and 1-, 2- and 3-year follow-up for the paediatric patients

Variables	Year 0	Year 1	Year 2	Year 3
Sex	*			
Date of birth	*			
Age at start of therapy	*			
Main diagnosis	*			
Grouped main diagnosis	*			
Additional diagnosis	*			
Grouped additional diagnosis	*			
Start days in hospital before discharge with LTMV	*			
Start situation at initiation of LTMV	*			
Acute versus elective initiation	*			
Treatment motive	*			
Type of treatment	*	*	*	*
Start of phrenic pacing	*	*	*	*
Prescribed duration (hour/day)	*	*	*	*
Additional equipment				
Oxygen (non-humidified, low flow)	*	*	*	*
Oxygen (humidified, high flow)	*	*	*	*
Humidifier	*	*	*	*
Suction device	*	*	*	*
Mechanical insufflator/exsufflator	*	*	*	*
Gastrojejunostomy or oesophageal tube	*	*	*	*
Nebuliser	*	*	*	*
Connection TIV versus NIV		*	*	*
Therapy terminated		*	*	*
Terminating date		*	*	*
Terminating cause		*	*	*
Ongoing therapy		*	*	*
Duration of therapy (days)		*	*	*

LTMV, long-term mechanical ventilation; NIV, non-invasive ventilation; TIV, tracheostomy invasive ventilation.

##### Swedish Intensive Care Registry (SIR)

The SIR is a national database established in 2001 to collect patient information from intensive care units across Sweden. It covers the entire country geographically and includes comprehensive data on diagnoses, medical procedures, hospital stays and patient-reported outcome measures.^34^

##### National Medical Birth Register (MBR)

The Swedish MBR covers approximately 98% of all births in Sweden. Since 1982, it has been compiled using prospectively collected data from standardised antenatal, delivery and neonatal care records. Once the mother and newborn are discharged from the hospital, this information is sent to the MBR, which undergoes yearly updates.^35^

##### Cause of Death Registry (DORS)

The Swedish DORS has been documenting all deaths in Sweden since 1952.^36^ A vast majority, up to 96% of individuals, have a clearly identified underlying cause of death recorded in the registry.^37^

##### National Patient Registry (NPR)

Information regarding comorbidities, hospitalisations, surgical and other medical procedures was obtained from the Swedish NPR, which encompasses both inpatient and outpatient care.^38^ The Inpatient Registry, also known as the Hospital Discharge Registry, was established in 1964 and achieved nationwide coverage in 1987. It includes almost all admissions to somatic and psychiatric hospitals, and since 2001, it has had nearly complete data coverage for public hospital-based outpatient services. However, data completeness for outpatient care is around 80%, as information from private healthcare providers and primary care is missing.^39^

##### Total Population Registry (RBT)

The RTB is kept by SCB. It is a demographic registry that dates back to the 1960s and contains data on dates of birth, sex, civil registrations and personal relationships. The coverage of the registry is high: it contains almost 100% of all births and deaths and between 91% and 95% of all immigrants arriving and emigrants leaving within the last 30 days.^40^

##### Longitudinal Integration Database for Health Insurance and Labour Market Studies (LISA database)

SCB provides demographic information for research purposes. The LISA database contains yearly data on various demographic factors, such as county and residence location, marital status, migration status, country of origin, educational attainment, employment status, income and health-related variables such as sick leave.^41^ LISA includes data on the adult population aged 16 years and older, registered as of December 31 each year since 1990, with an estimated completeness rate of 95%.^42^ The data collected include information about parents.

##### Registry for Interventions under the Act on Support and Service to Certain Disabled Persons (LSS registry)

The LSS registry^4^ is among the five social service registries managed by the National Board of Health and Welfare. Since 1999, the LSS registry has held personalised data concerning activities related to personal care assistance and care provision.^43^ In Sweden, personal care assistance is granted to individuals under 65 years of age who belong to one of the groups specified in the law, namely, those with developmental disabilities, significant intellectual disabilities caused by brain damage in adulthood or other permanent physical or mental disabilities not related to normal ageing that cause significant daily difficulties and necessitate extensive support or services.

Due to the governmental registries’ mandatory status, no patients will be lost to follow-up, except in cases of emigration. Details of the study population’s frequency of appearances in the included registries are described in table 2.

**Table 2:** Cross-tabulation of the Swedevox registry in relation to the National Quality Registry and mandatory Swedish governmental registries

	CPAP n=201	LTMV n=461	HFOT n=20	Other n=34	Main variables
National Patient Registry (NPR) n=700 (98) occurring patients	196	451	19	34	
Inpatient Registry n=16 748 care events	3910	11 707	387	744	Date of hospitalisation and discharge, hospital code, main diagnosis, secondary diagnosis(es), surgical diagnosis(es).
Outpatient Registry n=73 028 visits	19 773	48 282	1675	3298	Date of visit, hospital code, main diagnosis, secondary diagnosis(es), surgical diagnosis(es).
The Total Population Register (RTB) n=716 (100) unique patients	201	461	20	34	Date of birth, country of birth, Swedish or foreign background.
Registry for Interventions under the Act on Support and Service to Certain Disabled Persons (LSS registry) n=396 (57) unique patients	94	278	9	15	Presence of interventions from LSS, scope of interventions, presence of housing with special services.
Longitudinal Integration Database for Health Insurance and Labour Market Studies (LISA database) n=716 (100) unique patients	201	461	20	34	Family status, socioeconomic division, income and consumption weight for family.
National Medical Birth Register (MBR) n=511(71) unique patients	169	298	16	28	Date and time of birth, mode of delivery, singleton or multiple birth, gestational age, stillbirth, birth weight, birth length, head circumference, infant sex, Apgar scores, maternal and infant diagnoses/procedures, including neonatal care.
National Cause of Death Registry n=134 (19) deaths	13	111	4	6	Date of death, primary cause of death, contributing cause(s) of death.
Swedish Intensive Care Registry (SIR) n=480 (67) unique patients	117	316	18	29	Level of hospital (primary, secondary or tertiary), duration of hospitalisation, time of discharge, operation (Y/N, elective/acute), discharged alive (Y/N), diagnosis, decision to forego life-sustaining therapy (Y/N), mechanical ventilation (Y/N), cardiovascular, hepatic, gastroenterological, neurological, renal, respiratory, haematological, metabolic, traumatic or other reason for ICU care.

Data are presented as frequency (percentage).

Other: for example, tracheal cannula only and/or phrenic pacing.

CPAP, continuous positive airway pressure; HFOT, long-term high-flow oxygen therapy; LTMV, long-term mechanical ventilation.

### Patient and public involvement

A representative from the Swedish Heart and Lung Association patient organisation sits on the Swedevox registry’s steering committee and participated in discussions regarding study design.

## Findings to date

The findings reveal a group consisting of 716 children, 59% of whom are male, partly due to sex-linked diagnoses. The children commenced their respiratory support treatment at an average age of 6.4 (SD 5.4), with newborns and adolescents aged 11–16 years constituting 21% and 29% of the cohort, respectively. Among the participants, 201 (28%) are CPAP users, 461 (64%) are LTMV users, 20 (3%) are HFOT users and 34 (5%) use other methods, such as solely relying on a tracheal cannula and/or employing phrenic pacing or cough assistance for respiratory support. Time using respiratory support in the cohort was a median of 1650 days (IQR: 2496 days). Baseline characteristics stratified by ventilatory support modality are presented in table 3.

**Table 3:** Characteristics of the Course of DISease reported to the Swedish CPAP Oxygen and VEntilator RegistrY paediatrics (DISCOVERY-P) cohort

	CPAP n=201	LTMV n=461	HFOT n=20	Other n=34
Male sex, n	118 (58.7)	279 (60.5)	10 (50.0)	19 (55.9)
Age at start mean (SD)	6.7 (5.2)	6.3 (5.6)	3.3 (4.8)	6.0 (4.6)
Age at start median (IQR)	6 (10)	5 (11)	1.5 (4)	6 (7)
Age at start 0–1 years	50 (24.9)	154 (33.4)	10 (50.0)	8 (23.5)
Age at start 2–5 years	41 (20.4)	79 (17.1)	6 (30.0)	8 (23.5)
Age at start 6–10 years	48 (23.9)	85 (18.4)	1 (5.0)	11 (32.4)
Age at start 11–16 years	62 (30,8)	143 (31.0)	3 (15.0)	7 (20.6)
Phrenic pacing	0 (0.0)	0 (0.0)	0 (0.0)	3 (16.7)
Prescribed duration (hour/day)				
During sleep only (7–8 hours)	102 (82.3)	116 (45.8)	3 (18.8)	0 (0.0)
During sleep and some extra hours (<12 hours)	9 (7.3)	78 (30.8)	8 (50.0)	0 (0.0)
Major part of the day (12–24 hours)	6 (4.8)	15 (5.9)	4 (25.0)	7 (77.8)
Totally dependent (24 hours)	5 (4.0)	23 (9.1)	1 (6.2)	0 (0.0)
Daytime only	2 (1.6)	21 (8.3)	0 (0.0)	2 (22.2)
Oxygen (non-humidified, low flow)	14 (7.0)	62 (13.4)	5 (25)	1 (5)
Oxygen (humidified, high flow)	1 (0.5)	16 (3.5)	15 (75)	0 (0.0)
Humidifier	67 (33.3)	117 (25.4)	7 (35)	0 (0.0)
Suction device	24 (11.9)	75 (16.2)	8 (40)	10 (52.6)
Gastrojejunostomy or oesophageal tube	42 (20.9)	87 (18.9)	13 (65)	7 (20.6)
Nebuliser	32 (15.9)	58 (12.6)	1 (6.2)	0 (0.0)
TIV	3 (1.5)	74 (21.1)	4 (20.0)	11 (64.7)
NIV	177 (88.5)	271 (77.4)	15 (75.0)	3 (17.6)

Data are presented as frequency (percentage) or mean (SD) unless otherwise indicated.

Other: for example, tracheal cannula only and/or phrenic pacing.

CPAP, continuous positive airway pressure; HFOT, long-term high-flow oxygen therapy; LTMV, long-term mechanical ventilation; NIV, non-invasive ventilation; TIV, tracheal invasive ventilation.

The most common usage of respiratory support is at nighttime during sleep only, but a significant number of LTMV (54%) and HFOT (81%) users are also dependent on aid during the daytime. The study also shows that 77% of LTMV users rely on mask connection, compared with 66% reported internationally (2016–2023), indicating a difference in comparing Sweden to international practice.^13^ Some children use HFOT as an additional treatment and are reported as CPAP or LTMV with oxygen (humidified, high flow) as additional treatment.

Children using CPAP predominantly do so because of sleep apnoea (57%) often associated with craniofacial malformations or Mb Down. Those using LTMV do so primarily due to diseases affecting the brain or nervous systems (59%), although there are many variations, indicating a heterogeneous group. The complete distribution of primary diagnoses is outlined in table 4.

**Table 4:** Diagnosis distribution based on the main diagnosis

	CPAP n=201	LTMV n=461	LTOT n=20	Other n=34
Lung hypoplasia after diaphragmatic hernia	2	1	0	0
Other lung disease (BPD)	6	35	7	2
Spinal muscular atrophy Type I	1	16	0	1
Spinal muscular atrophy type II-III-IV	2	38	0	1
Duchenne muscular dystrophy	2	41	0	1
Other myopathies (limb girdle, nemaline myopathy, central core, Emery-Dreifuss…)	1	32	0	0
Myotonic dystrophy	0	13	0	1
Other congenital neuromuscular diseases; polyneuropathy (Charcot-Marie-Tooth, others)	1	48	0	3
Non-paralytic scoliosis/skeletal dysplasias	0	8	0	0
Arthrogryposis congenita	3	0	0	0
Myelomeningocele with thoracic deformity and/or Arnold-Chiari malformation	2	4	0	0
Chondrodystrophy/achondroplasia with hypoventilation	2	3	0	0
Spinal cord injury/transverse lesion	0	12	0	0
Cerebral palsy	17	20	1	0
Other brain injury (congenital or acquired)	18	63	7	4
Congenital central hypoventilation syndrome (CCHS)	0	12	0	12
Other central hypoventilation	1	5	0	2
Mitochondrial myopathy, neurometabolic disease	0	8	0	0
Prader-Willi	3	0	0	0
Degenerative brain diseases	0	2	0	1
Sleep apnoea with craniofacial malformation	19	2	0	0
Sleep apnoea with Down syndrome	34	6	1	0
Sleep apnoea with chondrodystrophy	1	0	0	0
Sleep apnoea other	60	17	0	1
Other or unspecified diagnosis	25	75	4	15

### Future plans

Future plans include investigating how socioeconomic factors influence hospitalisation rates, mortality and eligibility for personal assistance care, as well as exploring the relationship between the distance from home to the treatment centre and therapy adherence, including its impact on hospitalisation rates. These analyses will be conducted using both statistical modelling and subgroup comparisons to identify potential disparities in access to care. By addressing these questions, we hope to provide valuable insights into the factors shaping healthcare utilisation and outcomes for children requiring respiratory support. Society has both an opportunity and a responsibility to help these children and families to live healthy and dignified lives as part of the community. In order to bring about a community without health disparities, the outcomes and inequities in vulnerable groups such as children living with respiratory support need to be further studied.^44 45^ The DISCOVERY-P cohort, owing to its scale and comprehensiveness, stands poised to address significant knowledge gaps in research. The dataset is due for an update in 2026.

### Strengths and limitations

This nationwide population-based cohort stands out for its competitive size, extensive nationwide representation and remarkable data completeness. Numerous research studies based on data from the Swedevox registry have been previously published concerning the adult population,^3246^,^52^ and a comprehensive list of these publications is available in the annual report published by Swedevox.^10^

The cross-linkage with mandatory national registries ensures that no patients will be lost to follow-up. The Swedish system of using personal identity numbers facilitates the connection of individuals across various national registries, thereby enabling the creation of a distinctive database enabling the analysis of longitudinal data from diverse sources. However, it is important to acknowledge several limitations. To maintain a high level of completeness in the Swedevox registry, the number of variables requested for reporting has intentionally been kept limited, resulting in less detailed data. Moreover, the national quality registries lack mandatory status, resulting in somewhat varying coverage in both Swedevox and SIR. Challenges in adjusting for confounders represent a limitation common to all registry-based studies.

Throughout Sweden, 34 centres contribute patient data to the Swedevox registry; although the coverage is substantial, however, geographical gaps remain, leading to incomplete data. From 1 July 2023, long-term respiratory support for children has been classified as nationally highly specialised care,^53^ leading to the consolidation of care into four main centres catering to children requiring respiratory assistance (Skånes University Hospital, Karolinska University Hospital, Norrlands University Hospital and Salgrenska University Hospital).^54^ This consolidation is expected to enhance the comprehensiveness of future updates to the registry.

### Collaboration

We welcome and encourage contacts and requests for specific research projects and collaborative efforts, which can be directed to the corresponding author.

## Data Availability

Data are available upon reasonable request. Data may be obtained from a third party and are not publicly available.
