# Supplementary material for: Swedish national cohort of children living with long-term respiratory support (DISCOVERY-P): cohort profile
Source: BMJ Open. 2025 Apr 14;15(4):e090241. doi: 10.1136/bmjopen-2024-090241 (PMC11997812; doi:10.1136/bmjopen-2024-090241)
Supplement: Supplementary file 1 [file bmjopen-2024-090241.reviewer_comments.pdf]

PEER REVIEW HISTORY

BMJ Open publishes all reviews undertaken for accepted manuscripts. Reviewers are asked to complete a checklist review form and are provided with free text boxes to elaborate on their assessment. These free text comments are reproduced below.

ARTICLE DETAILS

Title (Provisional)

Cohort profile: Swedish national cohort of children living with long-term respiratory support (DISCOVERY-P)

Authors

Florén, Johan; Ekström, Magnus Per; Lindahl, B; Markstrom, Agneta; Palm, Andreas; Israelsson-skogsberg, Åsa

---

VERSION 1 - REVIEW

**Reviewer**	**1**
**Name**	**Barto, Tara**
**Affiliation**	**Baylor College of Medicine, Medicine**
**Date**	**13-Mar-2025**
**COI**	**None**

Summary: The paper is a cohort profile, description of an ongoing database development for pediatric patients requiring respiratory support aged 0-18 yrs. The paper reviews the different databases available to them in Sweden and how crosslinking them they can obtain nearly complete data. Descriptive analysis is presented of the data obtained to date. There is a comment on future directions.

Strengths:

1. I am impressed with the availability of data and the ability to cross link with other registries. I think this will provide a wealth of information for this population.

2. The authors described each of the databases clearly.

3. The authors reviewed appropriate descriptive statistics for this population

Weaknesses:

1. There was one comment on future plans but I would like to understand a little more what the authors plan to utilize this data and what questions they are planning to evaluate.

Specific Suggestions/Questions

1. In the abstract LTMV is used without previously defining it properly.

2. In the intro, 'fulfill' is misspelled

3. In reviewing the data in Table 3. It is odd to me that a high percentage of patients requiring HFOT than LTMV have tube feeding capability. I would think those requiring mechanical ventilation (59% of which are related to neurologic disease) would be more likely to require tube feeding support.

4. In reviewing table - it is odd to me the percentage of patients requiring suction or any nebuilzed therapy is quite low in the CPAP and LTMV population - this may be a function of practice in Sweden, but it seems again that those on HFOT are more likely to require suctioning than those on LTMV.

The last two points are just observations - it could be a function of how data is entered vs how patients are categorized, for example if someone uses HFOT during the day and CPAP at night - how would they be categorized?

Reviewer **2**

Name **Liu, Shijian**

Affiliation **Shanghai Children's Medical Center Affiliated to Shanghai Jiaotong University School of Medicine**

Date **14-Mar-2025**

COI **None**

The manuscript of bmjopen- 2024-090241 entitled "A Cohort profile of children (0–18) living with respiratory support reported to the Swedish CPAP Oxygen and Ventilator Registry", the cohort comprises of 716 children, 59% of whom are male, the topic is interesting, however some concerns existed and needed to be considered to improve for this manuscrpit.

Major points

1.Title: The title needs revising, I suggest to "Swedish National Cohort of Children on Long-Term Respiratory Support (DISCOVERY-P): Design, Baseline Characteristics, and Research Potentials";

2. Abstract: This study should emphasize the first nationwide cohort study, clinical and socioeconomic risk factors. Findings to date section, the author is encouraged to report some research findings beside basic description.

3. Introduction: It is lack of critical analysis of international research gaps on this fields.

4. Findings to Date: It is lack of statistical testing such as ANOVA (continuous variables) or χ^2 tests (categorical variables), $p<0.001$ for age differences, and clinical interpretation.

5. Table 2, please add “%” in the first row and corresponding results

Table 3, please delete “%” and add in the first row, add p-value column to compare differences between groups.

VERSION 1 - AUTHOR RESPONSE

Reviewer: 1. Dr. Tara Barto, Baylor College of Medicine, thank you for your thorough review. We hope the following changes and comments are well received.

1. There was one comment on future plans but I would like to understand a little more what the authors plan to utilize this data and what questions they are planning to evaluate.	Thank you for your curiosity, the text on future plans has been elaborated concerning methodology and questions. P16 Line 5-8
1. In the abstract LTMV is used without previously defining it properly.	Long-term mechanical ventilation (LTMV) is now updated. P2, Line 20.
2. In the intro, 'fulfill' is misspelled	The text is written in British English and fulfil is spelled accordingly.
3. In reviewing the data in Table 3. It is odd to me that a high percentage of patients requiring HFOT than LTMV have tube feeding capability. I would think those requiring mechanical ventilation (59% of which are related to neurologic disease) would be more likely to require tube feeding support.	Thank you for your insight. The data in the cohort are presented as reported to the registry.
4. In reviewing table - it is odd to me the percentage of patients requiring suction or any nebuilzed therapy is quite low in the CPAP and LTMV population - this may be a function of practice in Sweden, but it seems again that those on HFOT are more likely to require suctioning than those on LTMV.	The data in the cohort are presented as reported to the registry, reflecting clinical practice.
The last two points are just observations - it could be a function of how data is entered vs how patients are categorized, for example if someone uses HFOT during the day and CPAP at night - how would they be categorized?	Usage of CPAP in combination with high flow is categorised as CPAP with additional equipment, Oxygen (humidified, high flow). P 14 Line 6-7

Reviewer 2. Dr. Shijian Liu, Shanghai Children's Medical Center Affiliated to Shanghai Jiaotong University School of Medicine, thank you for your thorough review. We hope the following changes and comments are well received.

1.Title: The title needs revising, I suggest to "Swedish National Cohort of Children on Long-Term Respiratory Support (DISCOVERY-P): Design, Baseline Characteristics, and Research Potentials";	Thank you for the suggestion, new title has been updated.<br>P1 Line 1-3
2. Abstract: This study should emphasize the first nationwide cohort study, clinical and socioeconomic risk factors. Findings to date section, the author is encouraged to report some research findings beside basic description.	Wery good point, abstract has now been updated concerning it being the first nationwide cohort, as well as clinical and socioeconomic potential.<br>P2 Line 6-9<br><br>Owning to no further studies on the cohort has been published findings to date is limited.<br>P2 Line 18-23
3. Introduction: It is lack of critical analysis of international research gaps on this fields.	Gaps in international research has been addressed.<br>P4 Line 15-17
4. Findings to Date: It is lack of statistical testing such as ANOVA (continuous variables) or χ^2 tests (categorical variables), $p<0.001$ for age differences, and clinical interpretation.	Thank you for your valuable feedback. We have chosen not to include p-values in the table, as the purpose of our study is to describe the cohort rather than to perform statistical comparisons between groups. Conducting significance tests without a clear research question could lead to issues with multiple comparisons and mass significance. By focusing on a descriptive approach, we aim to present the data in a way that is both clinically relevant and methodologically appropriate.
5. Table 2, please add "%" in the first row and corresponding results<br>Table 3, please delete "%" and add in the first row, add p-value column to compare differences between groups.	Percentages have been added correspondingly in table 2.<br>P12<br>For table 3, "%" has been deleted, for p-value column se comment above.<br>P13
